# Factors influencing life satisfaction in acute myeloid leukemia survivors following allogeneic stem cell transplantation: a cross-sectional study

**DOI:** 10.1186/s12955-015-0222-8

**Published:** 2015-02-27

**Authors:** Susanne Amler, Maria Cristina Sauerland, Christian Deiters, Thomas Büchner, Andrea Schumacher

**Affiliations:** Institute of Biostatistics and Clinical Research, University of Muenster, Albert-Schweitzer-Campus 1, D-48149 Muenster, Germany; University Hospital of Muenster, Medicine A – Hematology, Oncology, Hemostaseology and Pneumology, Albert-Schweitzer-Campus 1, D-48149 Muenster, Germany

**Keywords:** Acute Myeloid Leukemia, Life satisfaction, Quality of life, Allogeneic stem cell transplantation, Oncology

## Abstract

**Background:**

Allogeneic stem cell transplantation (alloSCT) is the preferred option of postremission therapy for high-risk patients suffering from acute myeloid leukemia (AML). Therefore, monitoring life satisfaction (LS) of long-term survivors following alloSCT is becoming increasingly important for oncologists. The aim of the study was to evaluate individual survivor priority of various general and health-related domains of life and their satisfaction with these domains. Furthermore, we investigated the impact of general and health-related LS on resilience, anxiety, depression and quality of life in AML survivors following alloSCT.

**Methods:**

Forty-one AML survivors (median age at time of assessment = 49.0 years) who had undergone alloSCT (median time since transplantation = 3.1 years) were enrolled in the study. Psychosocial parameters were assessed using the following instruments: FLZ^M^ (Questions on Life Satisfaction), EORTC QLQ-C30, HADS (Hospital Anxiety and Depression Scale) and the RS-25 (Resilience Scale-25 items). Correlation analyses were computed to reveal the associations between the different questionnaires.

**Results:**

Independence from help or care, well-regulated living conditions and financial security contributed positively to LS, whereas being off work due to health-reasons and dissatisfaction with physical aspects were negatively associated to the subjective feelings of overall satisfaction. Moreover, a high quality of life was strongly positively correlated with LS (Spearman’s rho general LS: 0.643 and health-related LS: 0.726, both p < 0.001). A high degree of resilience was also strongly positively correlated with better LS (general LS: 0.700, health-related LS: 0.675, both p < 0.001). Symptoms of anxiety and depression were associated with an impaired general LS (anxiety: −0.674, depression: −0.698, both p < 0.001).

**Conclusions:**

Our results indicate that LS should be considered an important key contributor to the survivors’ well-being following alloSCT. Thus, identifying protective psychological and physical factors that relieve stressors is of high importance in order to support long-term AML survivors with their special needs.

## Background

Due to continuous optimization of chemotherapy and supportive care, the prognosis for patients suffering from acute myeloid leukemia (AML) has steadily improved over the last decades. As a consequence of these improvements, the quality of life of patients has become an important parameter to assess [1]. Aggressive treatment protocols can cause somatic late effects as well as psychosocial disorders [2-7]. Thus, a major challenge remains in the facilitation of an effective treatment that is also associated with a reliable, high quality of life.

To date, only a few studies have analyzed quality of life in AML survivors following alloSCT [8-10]. Messerer et al. demonstrated that due to a lack of studies concerning long-term follow-up, the relevance of the survivors’ quality of life often remains unclear. The authors postulated a significantly worse long-term impact of alloSCT on quality of life compared to conventional chemotherapy [11]. Grulke et al. analyzed quality of life before and after autologous or allogeneic haematopoietic stem cell transplantation from 33 publications which included about 2800 patients. Their results showed that quality of life decreased directly following transplantation, increased as date of discharge approached and continued to improve thereafter. Quality of life levels measured a year later were comparable to those measured a few weeks prior to transplantation [12].

The EORTC QLQ-C30 is the most widely used questionnaire in Europe that assesses quality of life in cancer patients [13]. Unfortunately, the advancement of research regarding the quality of life of adult AML survivors following alloSCT is slow [14]. It is possible that the EORTC QLQ-C30 core questionnaire provides only limited insight into the quality of life in cancer survivors. Kopp et al. reported that the EORTC QLQ-C30 affords insight that concerns mainly the physical aspects of quality of life and helps to identify symptoms, which decrease quality of life from the patients’ perspective [15]. However, some additional aspects of the quality of life concept still need to be addressed. Survivors’ self-assessed priority of and their satisfaction with various life domains often remain unclear [16]. The Questions on Life Satisfaction^Modules^ (FLZ^M^) is a validated questionnaire for assessing general and health-related quality of life [17]. The specific feature of this multidimensional construct is that it distinguishes between a person’s individual priority of multiple life domains and his/her own subjective satisfaction within each domain. The respondent evaluates each item twice, once for the subjective importance of the various aspects of general life and health conditions, and once for the degree of satisfaction in this domain. These two ratings are combined into a weighted satisfaction score. Presently, the multidimensional FLZ^M^ is a well-established tool used to measure LS in different oncological settings. [18-21]. To our knowledge, no study has yet been published on specific analysis of self-reported LS by using the FLZ^M^ in AML-survivors after alloSCT. We have therefore implemented the FLZ^M^ questionnaire, which provides a better understanding of survivors’ special needs.

Furthermore, psychological resilience, general living conditions and current life situations are rarely investigated. However, resilience could play an important role in addressing issues relating to the survivors´ satisfaction with life. Results of a study by Schumacher et al. revealed a strong correlation of resilience with quality of life [22].

Psychosocial care should no longer only focus on a limited number of psychological needs. In order to gain a better understanding of patients´ needs, it is important to identify a broader spectrum of psychological and social factors influencing patients´ well-being. Filtering key areas of need should be the first step to develop effective support care [23]. Using additional assessment tools, and thus gaining more extensive information concerning special issues, may help to develop a more accurate and systematic response to the urgent needs of individual patients.

Therefore, our cross-sectional study aimed at assessing LS by implementing the less commonly used FLZ^M^ questionnaire in long-term survivors who had received alloSCT and had been in complete remission for at least six months prior to evaluation. Since FLZ^M^ comparative data are available, we also compared our AML specific study sample with normative data from a representative German sample and with data from other cancer patients. In this way we expected to obtain a better insight into survivors’ importance rates and the satisfaction of their needs. Secondly, we investigated the association between LS within the FLZ^M^ and additional psychological factors such as resilience, anxiety, depression and quality of life by using other well-established questionnaires within the same study sample. In doing so we analyzed the ability of the FLZ^M^ to integrate with the other instrument tools, therefore enabling the use of the FLZ^M^ as an insightful and informative additional questionnaire. In addition, information on social issues, demographic and work-related variables was included. The findings of our study will help to identify important information regarding the special needs associated with AML cancer survivors after alloSCT.

## Methods

### Instruments

The following instruments were used: Questionnaire on Life Satisfaction FLZ^M^ [17] (in the original German: FLZ-M, Fragen zur Lebenszufriedenheit Module), Quality of Life Questionnaire EORTC QLQ-C30 [24,25], Resilience Scale RS-25 [26,27] and Hospital Anxiety and Depression Scale HADS [28,29]. The questionnaires are well-established evaluation tools used in psychological research.

In *Questions on Life Satisfaction FLZ*^*M*^, subjects assess their subjective general and health-related LS based on the subjective importance of certain aspects of multiple life domains and the satisfaction within these domains. Both modules consist of eight dimensions. The general module addresses the following: ‘friends/acquaintances’, ‘leisure time/hobbies’, ‘health’, ‘income/financial security’, ‘occupation/work’, ‘housing/living conditions’, ‘family life/children’ and ‘partner relationship/sexuality’. The health-related module addresses these issues: ‘physical condition/fitness’, ‘ability to relax/stay on an even keel’, ‘energy/zest for life’, ‘mobility (e.g., walking, driving)’, ‘vision and hearing’, ‘freedom from anxiety’, ‘freedom from aches and pains’ and ‘independence from help/care’. For each dimension, the assessment is completed in two steps: First, the subjects rate their subjective importance of each dimension on a scale of 1 (‘not important’) to 5 (‘extremely important’). Secondly, they assess their satisfaction with each dimension on a scale of 1 (‘dissatisfied’) to 5 (‘very satisfied’). The ratings for importance and satisfaction in each dimension are combined into a weighted satisfaction score by calculating the formula: *weighted satisfaction = (importance rating – 1) × (2 × satisfaction rating – 5)*. Possible dimension-specific weighted scores range from −12 to 20. Means and standard deviations were calculated for the weighted scores of each dimension. The two FLZ^M^ total scores, i.e. the general LS and health-related LS scores, are calculated by summing up the eight general and eight health-related scores. The total scores range from −96 to 160. Negative scores indicate dissatisfaction; positive scores indicate satisfaction of the specific dimension. The higher the score, the higher the subjective importance and individuals’ LS. Zero indicates no subjective importance [17]. The internal consistency as measure of the reliability for the FLZ^M^ General and FLZ^M^ Health total scores was shown to be high (Cronbach’s alpha = 0.82 and 0.89, respectively) [17,30]. In our study sample, internal consistency was also good with a Cronbach’s alpha of 0.83 for the general and 0.90 for the health-related module.

The EORTC Quality of Life Questionnaire (EORTC QLQ-C30 Version 3.0) consists of five functional scales (‘physical’, ‘role’, ‘emotional’, ‘cognitive’ and ‘social’). Three symptom scales, and six single items assess specific side effects. The ‘global quality of life scale’, consists of two items ‘How would you rate your overall health during the past week?’ and ‘How would you rate your overall quality of life during the past week?’ [24]. Raw scores are linearly transformed into scores ranging from 0 to 100, with a high score representing a higher response level. Thus, a high score in the functioning scales and the global quality of life scale indicates a high level of functioning, whereas a high score for a symptom scale or item reflects a high level of symptomatology [25]. The QLQ-C30 has been tested in EORTC filed studies [24] and is nowadays a very well validated instrument. In our study the reliabilities were acceptable or excellent: Cronbach’s alpha = 0.77 (physical), 0.86 (role), 0.90 (emotional), 0.86 (cognitive), 0.85 (social), fatigue (0.84), 0.93 (pain) and 0.89 (global scale). The reliability of fatigue was not comparable, because of missing values in our study sample.

The *Resilience Scale (RS-25) (German Version)* was originally developed by Gail M. Wagnild and Heather M. Young [27]. Items are scored on a seven-point Likert scale, ranging from 1 (‘strongly disagree’) to 7 (‘strongly agree’). The total score is calculated by summing up the 25 items of the resilience scale. Possible sum scores range from 25 to 175, with higher scores reflecting higher resilience [27]. Internal consistency of the German version of the RS-25 Scale [26] was evaluated in a large community sample of the German population (n = 2031, aged 4–95 years) with a high Cronbach’s alpha of 0.95. In the current study, Cronbach’s alpha was 0.91 for the whole scale.

The *Hospital Anxiety and Depression Scale (HADS) (German Version)* [29] was originally developed by Zigmond and Snaith [28]. Each of the 14 question items, seven for each subscale, is a four point (0–3) category. The overall possible sum scores range from 0 to 21 for anxiety as well as for depression. Lower scores (0–7) indicate clinical stability; whereas higher positive scores (greater or equal to 11) suggest potential need for psychiatric treatment.

The well-validated German version of the HADS showed acceptable internal consistencies for both scales [29,31]. In our study, internal consistency was good (Cronbach’s alpha of 0.86 for both the depression and anxiety scale) and excellent for the whole scale of the HADS: Cronbach’s alpha = 0.92.

Clinical data from survivors such as type of AML, age at diagnosis and randomized induction regime were provided by the central database of the study office. In addition, general data about survivors’ current life situation were collected, including socio-demographic characteristics such as age, sex, education, income, marital status and actual size of household, respectively.

### Setting and population

This quality of life study is based on a subgroup of AML survivors who had previously undergone treatment according to protocol of the multicenter clinical AML Cooperative Group (AMLCG 99) trial for newly diagnosed primary AML [32]. Inclusion criteria for the quality of life study were: 1. AML patients were 16*–*60 years of age at time of initial diagnosis and had already received conditioning therapy. 2. Patients achieved first complete remission following conditioning treatment and 3. Subsequent alloSCT was performed at least 6 months prior to assessment. Taking these criteria into account, 84 eligible patients were deemed suitable for quality of life assessment. These patients were treated at 27 various German cancer centers. They received transplantation between December 1999 and August 2005. We knowingly accepted the relatively long period between time of transplant and assessment due to the pilot character of the study. The AMLCG 99 trial was approved by the Ethics Committee of the University of Muenster (Muenster, Germany) and was conducted in accordance with the ethical standards established in the 1964 Declaration of Helsinki and its later amendments. Survivors meeting inclusion criteria for the quality of life study were informed by clinicians from their respective clinical centers. Written informed consent was given by all survivors prior to trial inclusion. The quality of life assessment was conducted in an outpatient setting.

### Data collection

Data were collected from June 2006 to March 2007. One patient was untraceable due to unknown address. Three patients were not contacted due either to serious psychological difficulties, premature withdrawal, or relapse. Therefore, the questionnaires including a separate information letter regarding the quality of life study were sent to the remaining 80 patients with a pre-addressed, pre-paid, return postage label. Survivors expressed their consent by voluntarily completing the questionnaires and returning them via post to the study office. 41 survivors complied, representing a response rate of 51%.

### Data analyses

Data collection, transformation and the statistical analyses of the data were performed using IBM SPSS Statistics 20.0 for Windows (IBM Corporation, Somers, NY, USA) and SAS software, Version 9.2 (SAS Institute Inc., Cary, NC, USA).

To examine a potential effect between LS and assessment on global quality of life, resilience, anxiety and depression, rank correlations were calculated using the method of Spearman [33]. Correlation coefficients were interpreted using the scale provided by Salkin, where an r between 0.8 and 1.0 is defined as ‘very strong’, between 0.6 and 0.8 as ‘strong’, between 0.4 and 0.6 as ‘moderate’, between 0.2 and 0.4 as ‘weak’ and between 0.0 and 0.2 as ‘very weak’ or ‘no relationship’ [34]. In order to guarantee comparability with normative data and other samples, group comparisons regarding the FLZ^M^ total scores are presented as mean with standard deviation, otherwise medians with interquartile ranges (IQR) are presented.

Nevertheless, in order to do justice to that effort with the relatively small study sample size, FLZ^M^ total scores (e.g. general LS, health-related LS) were compared between categorical groups using non-parametric Mann–Whitney U test [35,36] for two group comparisons, whereas the Kruskal-Wallis test was performed on three or more groups [37]. Differences between proportions were analyzed using Fisher’s exact test [38]. Inferential statistics are intended to be exploratory (hypotheses generating), not confirmatory, and are interpreted accordingly. P-values are considered statistically significant in case p < 0.05.

## Results

### Study sample

We identified a total of 41 AML survivors, aged 23–66 years at time of assessment, who fulfilled the questionnaires. Among those, 44% were men (n = 18) and 56% of the participants were women (n = 23). With regard to age at time of assessment, there were no significant age differences between men and women (median: 47.0 vs. 49.0, p = 0.636; data not shown). At the time of diagnosis, 66% of the participants had de novo AML (n = 27), 29% suffered from secondary AML (n = 12) and 5% had high-risk myelodysplastic syndrome (n = 2). Participating survivors were followed up with a median of 3.1 years post alloSCT (ranged from 8 months to 7 years).

Table 1 summarizes the characteristics in a comparison between participants and survivors who declined participation in the quality of life study. There were no significant differences between participants and non-participants regarding sex, age at diagnosis, type of conditioning treatment, donor or occurrence of graft-versus-host disease. Differences were found between the two groups regarding type of hematological disease: a higher proportion of participants than non-participants had secondary AML and high-risk myelodysplastic syndrome (p = 0.022). Its participating survivors were slightly older than the survivors who declined participation (median: 49.0 vs. 44.0; p = 0.046).

**Table 1:** **Characteristics of the AML survivors (n = 80)**

	**Survivors contacted for the quality of life study**	**Participating survivors (P)**	**Survivors who declined participation (NP)**	***p*** **-value**
				**P vs. NP**
**No of survivors**	80	41	39	
**Sex**, N (%)				0.509
Male	38 (47)	18 (44)	20 (51)	
Female	42 (53)	23 (56)	19 (49)	
**Age at diagnosis** (years)	42.0	43.0	40.0	0.070
Median (IQR^b^)	(34.0 − 49.0)	(37.0 − 51.0)	(32.0 − 47.0)	
**Age at time of assessment** (years)	45.5	49.0	44.0	0.046
Median (IQR)	(38.0 − 52.5)	(41.0 − 54.0)	(38.0 − 49.0)	
**Type of AML**, N (%)				0.022
De Novo AML	62 (77)	27 (66)	35 (90)	
Secondary AML^a^	15 (19)	12 (29)	3 (8)	
High-risk myelodysplastic syndrome	3 (4)	2 (5)	1 (3)	
**Induction therapy**, N (%)				0.509
TAD-HAM	38 (47)	18 (44)	20 (51)	
HAM-HAM	42 (53)	23 (56)	19 (49)	
**Time interval since alloSCT** (years)	3.3 (2.4 − 4.8)	3.1 (2.4 − 4.2)	3.7 (2.5 − 5.3)	0.191
Median (IQR)				
**Donor**, N (%)				0.062
Related	58 (72)	26 (63)	32 (82)	
Unrelated	22 (28)	15 (37)	7 (18)	
**HLA** ^**c**^, N (%)				0.258
Matched	73 (91)	39 (95)	34 (87)	
Mismatched	7 (9)	2 (5)	5 (13)	
**GvHD** ^**d**^ **after transplantation**, N (%)				1.000
Yes	52 (68)	26 (67)	26 (68)	
No	25 (32)	13 (33)	12 (32)	

^a^Secondary AML included AML after myelodysplastic syndrome or therapy-related AML.

^b^IQR = Interquartile range.

^c^HLA = human leukocyte antigen. ^d^GvHD = graft-versus-host-disease.

### Survivors’ general and health-related LS

**Figure 1:**
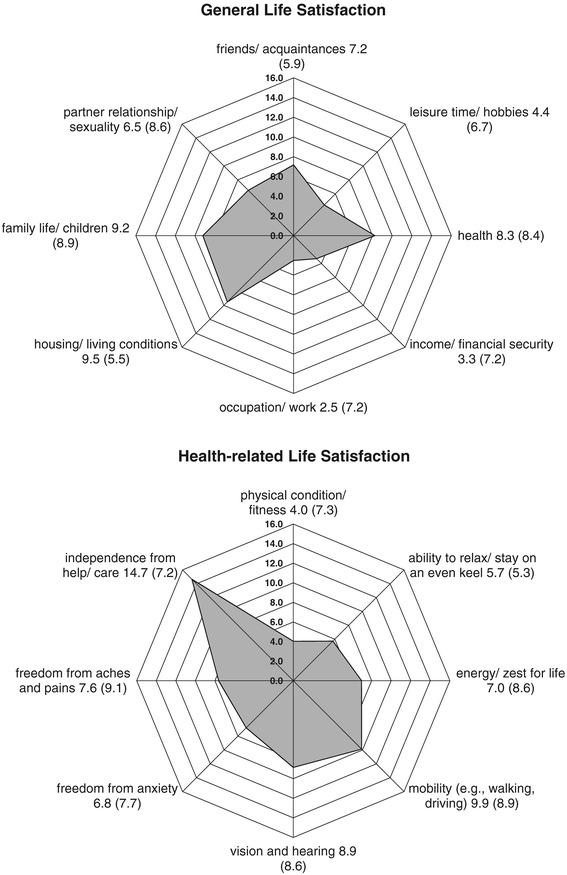
**Life satisfaction as measured by the FLZ** ^**M**^ **.** Group means with 1-fold standard deviations (displayed in brackets) are reported (n = 41). The distances from the center point indicate the weighted scores of each item. The marked area enclosed between the eight items represents the total general or health-related life satisfaction within the study group.

Figure 1 shows the weighted LS mean scores of the study population for the eight different dimensions, separated by the general and health-related modules. Analysis of the general life domains showed that the study participants were most satisfied with their ‘housing/living conditions’ (mean = 9.5), closely followed by ‘family life/children’ (mean = 9.2) and ‘health’ (mean = 8.3). By contrast, the minimum weighted LS value were found for ‘occupation/work’ (mean = 2.5), followed by ‘income/financial security’ (mean = 3.3). When investigating the different dimensions of the health-related module, participants were most satisfied with their ‘independence from help/care’ (mean = 14.7), followed at some distance by ‘mobility’ (mean = 9.9) and ‘vision and hearing’ (mean = 8.9). Here, the participants were less satisfied with their ‘physical condition/fitness’ (mean = 4.0), followed by ‘ability to relax/stay on an even keel’ (mean = 5.7).

### Correlations between the FLZ^M^ and other questionnaires

Correlations between weighted general and health-related LS and the other parameters were computed. The global quality of life scale (2-item) of the QLQ-C30 showed a strong correlation with survivors’ weighted LS (Spearman’s rho r = 0.643 for general LS and r = 0.726 for health-related LS; both p < 0.001). Furthermore, there was a strong positive correlation between ‘emotional functioning’ and the general LS (r = 0.687) and a moderate correlation with the health-related LS (r = 0.564), both p < 0.001. ‘Physical-’, ‘cognitive-’ and ‘role functioning’ correlated moderately with survivors’ LS for both FLZ^M^ total scores (general LS: r = 0.529, r = 0.539, r = 0.580; all p < 0.001; health-related LS: r = 0.599, r = 0.545, r = 0.554; all p < 0.001). ‘Social functioning’ also correlated moderately with general LS (r = 0.515; p < 0.001) but correlated weakly with health-related LS (r = 0.244; p > 0.05). HADS anxiety and depression scores showed a strong negative correlation with survivors’ general LS (anxiety: r = −0.674, depression: r = −0.698; both p < 0.001). A similar negative effect was seen for correlation with health-related LS (anxiety: r = −0.536, depression: r = −0.739; both p < 0.001). In contrast, a strong positive correlation was found for LS with resilience (general LS: r = 0.700, health-related LS: r = 0.675; both p < 0.001). Bivariate correlation coefficients for the general LS and health-related LS total scores within the FLZ^M^ and the other instruments are summarized in Table 2.

**Table 2:** **Correlations (Spearman’s rho): life satisfaction (FLZ** ^**M**^ **), functioning scales of EORTC QLQ-C30, resilience scale RS-25 and HADS**

	**General LS**	**Health-related LS**	**Global quality**	**Physical functioning**	**Emotional functioning**	**Cognitive functioning**	**Role functioning**	**Social functioning**	**Resilience scale**	**HADS-D-anxiety**
Health-related life satisfaction	0.711**									
Global quality of life (QOL)	0.643**	0.726**								
Physical functioning	0.529**	0.599**	0.781**							
Emotional functioning	0.687**	0.564**	0.647**	0.591**						
Cognitive functioning	0.539**	0.545**	0.511**	0.374*	0.509**					
Role functioning	0.580**	0.554**	0.681**	0.601**	0.626**	0.598**				
Social functioning	0.515**	0.244	0.403**	0.436**	0.635**	0.377*	0.408**			
Resilience Scale	0.700**	0.675**	0.538**	0.495**	0.713**	0.513**	0.488**	0.409**		
HADS-D-Anxiety	−0.674**	−0.536**	−0.525**	−0.526**	−0.828**	−0.487**	−0.438**	−0.559**	−0.722**	
HADS-D-Depression	−0.698**	−0.739**	−0.751**	−0.609**	−0.699**	−0.577**	−0.594**	−0.493**	−0.695**	0.720**

*p < 0.01. **p < 0.001.

### Sociodemographic and medical characteristics associated with the survivors’ LS

The association between sociodemographic and clinical characteristics was investigated for both general LS and health-related LS (Table 3). The largest association was observed for employment status. Survivors who had retired for health reasons or were currently on sick leave had significantly worse health-related LS (p = 0.033). Moreover, in secondary AML the average scores for general LS and health-related LS were noticeable lower (33.8 and 44.9, respectively) than in de novo AML (57.8 and 69.5, respectively). Not surprisingly, younger people tend to have better health-related LS and, as can be expected, decline with growing age. No significant effects on LS were found for the other sociodemographic factors.

**Table 3:** **FLZ** ^**M**^ **-general LS and FLZ** ^**M**^ **-health LS total scores for the AML survivors study sample by sociodemograhic and clinical factors**

	**n** ^*^	**FLZ** ^**M**^ **general LS**	***p*** **-value**	**FLZ** ^**M**^ **health**	***p*** **-value**
**Total**	40	50.7 (39.1)		64.9 (48.0)	
**Age at diagnosis** (years)	40	0.645 [−0.612;1.902]^a^	0.305^b^	−0.412 [−1.959; 1.136]^a^	0.593^b^
**Age at time of evaluation** (years)	40	0.601 [−0.635;1.838]^a^	0.331^b^	−0.610 [−2.127; 0.907]^a^	0.420^b^
**Time interval since alloSCT** (years)	40	−0.081 [−8.409;8.247]^a^	0.984^b^	−6.338 [−16.197;3.521]^a^	0.201^b^
**Sex**			0.542		0.702
Male	18	46.3 (50.6)		59.4 (54.3)	
Female	22	54.1 (28.2)		67.5 (43.1)	
**Type of AML**			0.272		0.096
De Novo AML	27	57.8 (38.4)		9.5 (51.5)	
Secondary AML	11	33.8 (41.1)		44.9 (37.8)	
High-risk myelodysplastic syndrome	2	39.0 (15.6)		92.0 (0)	
**Marital status**			0.466		0.717
Not married	9	37.1 (47.4)		66.6 (47.5)	
Married	24	52.0 (39.9)		56.8 (54.3)	
Separated/Divorced/Widowed	7	63.0 (28.4)		80.3 (22.7)	
**Employment status**			0.238		0.033
Full-time	9	65.6 (30.7)		86.8 (24.2)	
Part-time/Marginal	6	57.2 (47.5)		86.5 (38.2)	
Currently off work due to illness	4	57.5 (30.7)		37.5 (68.3)	
Retired for health reasons	11	24.8 (44.9)		25.8 (48.6)	
Unemployed/Other	8	58.6 (35.2)		76.9 (35.1)	
**Educational level**			0.099		0.070
Low (elementary school)	21	41.9 (43.1)		50.7 (46.4)	
Middle (secondary school)	10	74.5 (27.2)		82.9 (52.4)	
High (grammar school)	4	30.5 (34.5)		52.5 (58.9)	
University	4	57.0 (35.3)		91.0 (8.8)	

*Sample size varies due to missing or incomplete data.

^a^Regression coefficient beta with 95% confidence interval.

^b^p-value determines whether or not the null hypothesis that a particular predictor's regression coefficient is zero can be rejected.

Note: unless otherwise specified, means with 1-fold standard deviation are reported.

## Discussion

As scientific and medical progress in alloSCT are likely to increase the number of long-term survivors, these survivors are faced with lasting physical and psychosocial problems related to transplantation. In order to offer each individual patient the maximum physical and psychological advantage following this intensive therapy, it is important to examine and identify protective psychosocial factors [22]. The survivor’s individual priority of various life domains and their satisfaction with these domains often remain unclear. This necessitates the need to know in detail how satisfied a person is with a specific aspect of life, as well as how important this aspect is to that person. Therefore, the main focus of our study was the assessment of LS in long-term AML survivors who underwent alloSCT.

In our study, we detected different levels of survivors’ importance and satisfaction for a variety of life domains. In terms of the general LS, we found the largest values in ‘housing/living conditions’ and ‘family life children’. This not only means that housing situation and family structure are of great importance for the majority of the AML survivors, but that they also report a very high satisfaction in these two specific life domains. The lowest values were found for the domains ‘income/financial security’ and ‘occupation/work’. This ranking seems to confirm that being employed and ensuring a good steady income are one of the most important aspects of life, yet a high proportion of the study group are dissatisfied with their employment conditions. Differences in health-related LS were also reflected when comparing the occupational status among SCT survivors: unemployment due to illness or retirement for health reasons had a significantly negative impact on health-related LS. Our finding is in line with a study from Sweden concerning adult survivors with a former hematological disease and a median follow up of 8 years post alloSCT [39]. The authors stated that being employed is an important goal after alloSCT. In general, employment is associated with better well-being among survivors previously treated with hematopoietic SCT [40]. Other studies also show that the most prevalent problems among SCT survivors included concerns about keeping their job, worries that they will need to take a disability pension or a paid leave of absence [41]. This financial and employment stress leads many survivors to a poorer quality of life [40,42-44]. It should be noted that more than half of the participants (56%) of our study sample were unemployed at time of follow up which may explain the great impact on the survivors’ dissatisfaction with their financial status. Moreover, domains of the health-related LS showed a high degree of satisfaction among ‘independence from help/care’ and ‘mobility’, whereas survivors’ LS was most negatively affected by ‘physical condition/fitness’. However, physical fitness limitations are a commonly known side effect, especially among survivors following SCT [45-47]. Other studies noted that a substantial percentage of AML patients had difficulties with physical activities. In Zittoun et al. 49% out of a total of 35 alloSCT patients in complete remission still reported difficulties in taking a long walk or doing work or household jobs [7]. Furthermore, it is not surprising that survivors are not completely satisfied in the domains that reflect psychological components, such as ‘ability to relax/stay on an even keel’ , ‘freedom from anxiety’, ‘energy/zest for life’ and ‘freedom from aches and pains’. This may reflect the survivors’ greater risk for symptoms of anxiety and depression following an aggressive cancer therapy. Consistent with previous studies assessing the impact of psychological distress on quality of life for hematological cancer survivors, higher levels of psychological morbidity (e.g. anxiety and depression) were associated with survivors reporting lower satisfaction with health [40,48,49].

In direct comparison to a study with a representative sample of the German population by Henrich and Herschbach [17], in our study sample the FLZ^M^ general LS total scores were on average about 10 scores lower than the representative sample (mean with standard deviation, sample size): 60.5 (37.3, n = 2534) vs. 50.7 (39.1). For the FLZ^M^ Health LS the total scores were equally lower in the study sample than in the representative sample: 74.4 (41.5) vs. 64.9 (48.0). As of yet, there are no directly comparable values for the FLZ^M^ in survivors suffering from AML following alloSCT. With regard to the specific eight life domains, in both the representative and the study sample, the highest impacts were observed for ‘housing/living condition’ and ‘family life/children’ and the lowest values in the domain ‘occupation/work’ [17]. A similar effect can also be observed for the eight FLZ^M^ Health modules with highest value for ‘independence from help/care’ and lowest values for ‘physical condition/fitness’ and ‘ability to relax’ in both samples [17].

When comparing our results with the data from other cancer patients, patients under treatment scored on average slightly lower in the FLZ^M^ General LS [17]. In contrast, those cancer patients in rehabilitation or in remission assessed their life satisfaction as higher than the participants in our study.

Overall, our study sample of AML survivors following alloSCT are not as satisfied with their subjective life satisfaction as the representative sample, but are more satisfied than acute cancer patients currently under treatment.

The correlation of the FLZ^M^ with the other instruments demonstrates a strong or moderate positive effect of quality of life, including all functional scales of the EORTC QLQ-C30 and of the psychological well-being on survivors’ LS. In contrast, the subscales anxiety and depression of the HADS, revealed a strong negative impact on survivors’ LS. These results are in accordance with other studies, in which a positive correlation was found between resilience and LS, and a negative correlation with psychological stressors [16,50,51].

However, with respect to survivors who were contacted, yet decided not to respond, the reasons for not participating in the quality of life study are unclear. It is possible that some of the long-term survivors no longer wanted to be confronted with their former disease. Some of them might have moved on with their lives. In fact, we had detailed information from one non-responder that he no longer wanted to think intensively about his former disease. Response rates might also be dependent on the centers’ specific size and specialization. Interestingly, the survivors responding to the questionnaires were older at time of assessment and had a higher risk profile (e.g. higher age, higher rate of transplantations from unrelated donors, less de novo AML and more secondary AML and high-risk myelodysplastic syndrome). Thus, it is possible that these survivors, having a generally lower quality of life, may be more motivated to answer questionnaires. Compared to older survivors, younger survivors may have been less likely to respond, due to different priorities in terms of families and job duties, which is in line with a study by Hall et al., suggested that younger survivors may be more likely to have competing time demands [43].

Despite these possible explanations, all survivors were in complete remission at the time of questionnaire evaluation, and there were no significant differences in treatment-related characteristics between participants and non-participants. It should be noted, however, that our findings are restricted due to the cross-sectional design of our study. Furthermore, although the sample size was rather small for an assessment, this can be explained by the fact that being a rare disease, the recruitment of former AML patients is a limiting factor. The overall response rate was more than 50%.

### Conclusion and future implications

In order to adequately support long-term survivors with special needs, physicians and psychologists need to look carefully at survivors’ satisfaction with life and the subjective importance of specific life domains.Whereas the assessment of quality of life is increasingly common in studies, it often remains unclear how important and satisfied a specific life domain is for an individual patient. Our study indicates that the FLZ^M^ questionnaire corresponds well with other validated instruments. Our findings emphasize that the use of the FLZ^M^ provides important information, which offers a broader spectrum and more precise details of survivors’ quality of life. This allows us to reach a clearer understanding of how best to identify the needs of AML survivors and will help to address such needs. Our pilot study has shown that LS in AML survivors following alloSCT is an important issue and its assessment provides valuable information and insights that affect future research. Since our study showed a substantial lack of satisfaction in terms of employment issues, physicians and psychologists should also focus their efforts on areas such as financial support services. A good example would be providing assistance for reintegration into social and professional life. Moreover, it is very important to offer interventions at the earliest possible stage of clinical care that could prevent psychological distress, and physical limitations could be addressed in the form of special fitness programs following alloSCT.

## Abbreviations

AML: Acute myeloid leukemia
AlloSCT: Allogeneic stem cell transplantation
LS: Life satisfaction
